# Effects of isoflurane, remifentanil and dexmedetomidine on selected EEG parameters derived from a Narcotrend Monitor before and after nociceptive stimulation at different MAC multiples in cats

**DOI:** 10.1186/s12917-020-02532-y

**Published:** 2020-09-14

**Authors:** Jonathan F. Raue, Julia Tünsmeyer, Sabine B. R. Kästner

**Affiliations:** 1 Small Animal Clinic, University of Veterinary Medicine Hannover, Foundation, Bünteweg 9, 30559 Hannover, Germany; 2 Center for Systems Neuroscience Hannover, University of Veterinary Medicine Hannover, Foundation, Bünteweg 9, 30559 Hannover, Germany

**Keywords:** Anaesthesia, Anaesthetic depth, Cat, Dexmedetomidine, EEG, Electroencephalography, Isoflurane, Minimum alveolar concentration, Narcotrend, Remifentanil

## Abstract

**Background:**

The aim of this prospective and complete cross-over study was to evaluate the effects of isoflurane, remifentanil and dexmedetomidine on EEG parameters derived from the Narcotrend® Monitor before and after nociceptive stimulation at different isoflurane MAC (minimal alveolar concentration) multiples. Seven adult European Domestic Short Hair cats were used. Each cat went through 3 experimental treatments. Group I received isoflurane, group IR received isoflurane and a constant rate infusion (CRI) of remifentanil (18 μg/kg/h IV), and group ID received isoflurane and a CRI of dexmedetomidine (3 μg/kg/h IV). The isoflurane MAC in each group was determined via supramaximal electrical stimulation. The EEG parameters were derived by a Narcotrend Monitor at specific time points before and after nociceptive stimulation at 0.75, 1.0 and 1.5 MAC.

The depth of anaesthesia was also assessed by a clinical score.

**Results:**

The mean MAC sparing effects in group IR and group ID were 9.8 and 55.2%, respectively. The best correlation of EEG and MAC multiples was found for the Narcotrend Index (NI) in group I (r = − 0.67). The NI was also able to differentiate between 0.75 MAC and 1.5 MAC in group IR. Spectral edge frequency had a lower correlation with MAC multiples in group I (r = − 0.62) but was able to differentiate between 0.75 MAC and 1.5 MAC in groups I and IR, and between 1.0 MAC and 1.5 MAC in group IR. Narcotrend Index, SEF 95 and MF increased significantly after nociceptive stimulation at 1.0 MAC in group I, and SEF 95 increased significantly at 0.75 MAC in group ID. The clinical score correlated closer than any of the EEG parameters with MAC in all groups, with highest correlation values in group I (r = − 0.89). Noxious stimulation led to a significant increase of the clinical score at 0.75 MAC and 1.0 MAC in group I.

**Conclusions:**

The EEG parameters derived from the Narcotrend Monitor show correlation to isoflurane MAC multiples in cats, but the anaesthetic protocol and especially the addition of dexmedetomidine have great influence on the reliability. The Narcotrend Monitor can be used as an additional tool to assess anesthetic depth in cats.

## Background

Since unconsciousness is a main target of general anaesthesia, electroencephalography has awakened increasing interest in terms of assessment of anaesthetic depth during the last four decades, in humans as well as in other species [1–3]. The parameters measured by surface electrodes on the skull provide informations about the electrical activity of the cerebral cortex, which is influenced by several factors including anaesthetic depth, anaesthetic drugs, and physiologic parameters.

Because the interpretation of raw EEG data is difficult without special knowledge and also time-consuming [4], more application-oriented solutions such as the BIS, CSM and Narcotrend Monitor [5–9] have been invented. They use special algorithms for processing the raw EEG data, thereby allowing real-time interpretation and providing the anaesthesiologist with clinically useful information. The BIS and the CSM have already been evaluated as possible tools for assessing anaesthetic depth in dogs and cats [10–13]. The Narcotrend Monitor’s algorithms, which are based on a visual classification of human sleep EEG, spectral parameters and the detection of burst suppression lines [14], are able to identify specific sleep EEG patterns which are then processed and result in the displayed dimensionless Narcotrend Index (NI), ranging from 0 (electrical silence) to 100 (awake). This index corresponds to six displayed EEG stages, ranging from A to F, with 15 substages.

Multiple studies have been performed regarding the Narcotrend Monitor’s potential usefulness in human anaesthesia, with varying results, depending on the setting and the anaesthetic protocol [5–7, 15]. However, in veterinary medicine, there are only few studies which evaluate anaesthetic monitoring via Narcotrend, including species such as horses [16] and dogs [17, 18] with different anaesthetic protocols.

To the author’s knowledge, there is no information available on the Narcotrend Monitor used for feline anaesthetic monitoring. Therefore, the aim of this study was to evaluate the influence of several anaesthetic protocols and noxious stimulation on the Narcotrend EEG parameters at different isoflurane MAC multiples in cats, and to compare the results with a clinical score of anaesthetic depth.

## Results

One cat was excluded from group ID due to the development of ongoing 2nd degree atrioventricular blocks and low blood pressure after starting the dexmedetomidine CRI. The blocks disappeared after disconnecting CRI. This cat did not show any signs of arrhythmia in the other experimental groups. All recordings from the other 6 cats were free of arrhythmia.

The mean ± SD isoflurane 1.0 MAC values were 1.83 vol% (SD ± 0.22 vol%), 1.65 vol% (SD ± 0.13 vol%) and 0.82 vol% (SD ± 0.2 vol%) in group I, IR and ID, respectively. This resulted in a significant MAC sparing effect of 55.2% (*p* = 0.003) in group ID, but not in group IR. In group IR, two cats had higher individual MAC values compared to their individual MAC in group I.

Comparing the prestimulation values (Additional file 1) within the treatment groups, SEF was different between 0.75 MAC and 1.5 MAC in group I (*p* = 0.02) and in group IR (*p* = 0.01), as well as between 0.75 MAC and 1.0 MAC in group I (p = 0.02) and between 1.0 MAC and 1.5 MAC in group IR (p = 0.01). Alpha band presence was significantly different between 0.75 MAC and 1.5 MAC in group IR (*p* = 0.0272), and θ band presence was significantly different between 0.75 MAC and 1.0 MAC (*p* = 0.0207) in group IR, as well as between 0.75 MAC and 1.5 MAC (p = 0.0207) in the same group. In group ID, θ band presence was different between 1.0 MAC and 1.5 MAC (*p* = 0.0289). The NI was significantly different between 0.75 MAC and 1.5 MAC in group IR.

As shown in Additional file 1, none of the measured EEG parameters was able to detect noxious stimulation by showing significant differences between pre- and post-stimulation values at more than 1.0 MAC multiple. The NI increased after stimulation significantly only in group I at 1.0 MAC (*p* = 0.0469). The α band presence decreased significantly in group IR at 1.0 MAC (p = 0.0469), θ band presence decreased significantly in group I at 1.0 MAC (*p* = 0.0313) and in group ID at 1.5 MAC (p = 0.0313). δ band presence decreased significantly in group ID at 1.5 MAC (p = 0.0313). The SEF increased in group I at 1.0 MAC (p = 0.0313) and in group ID at 0.75 MAC (p = 0.0313) with stimulation. In group I, MF decreased significantly at 1.0 MAC (*p* = 0.0469).

At 0.75 MAC, Power was significantly lower in group ID than in group IR (*p* = 0.0295). The prestimulation NI was significantly higher in group IR than in group I at 1.0 MAC (*p* = 0.0291). Spectral edge frequency values were significantly higher in group ID compared with groups I (*p* = 0.014) and IR (*p* = 0.0112) at 1.5 MAC.

Also at 1.0 MAC, the pre−/poststimulation difference value of the β band was significantly higher (p = 0.014) in group ID than in group I, and the difference values of the θ band of group ID (*p* = 0.0081) and group IR (*p* = 0.0057) were significantly lower than in group I. At 1.5 MAC, α band presence was lower in group ID than in group IR.

At 1.0 MAC, the difference value of the θ band in group I was significantly higher than at 0.75 MAC and 1.5 MAC (*p* = 0.0207). For β band (*p* = 0.012) and spectral edge frequency (*p* = 0.0289) at 1.5 MAC, the pre−/poststimulation difference values were significantly lower in group ID compared with 0.75 MAC.

In group I, burst suppression patterns were found in one cat in the pre-stimulation phase at 0.75 MAC and 1.5 MAC, and in 2 cats at 1.0 MAC. In group IR, this pattern was obvious in the pre-stimulation phase of one cat at 1.0 MAC, and in group ID, one cat did show burst suppression patterns at 1.5 MAC in the pre- and post-stimulation phase. Isoelectricity was found in the pre- and post-stimulation phase in several cats in groups I and IR at 1.5 MAC but not in group ID. At lower MAC multiples EMG activity was evident in all groups, with increased activity after noxious stimulation.

**Fig. 1:**
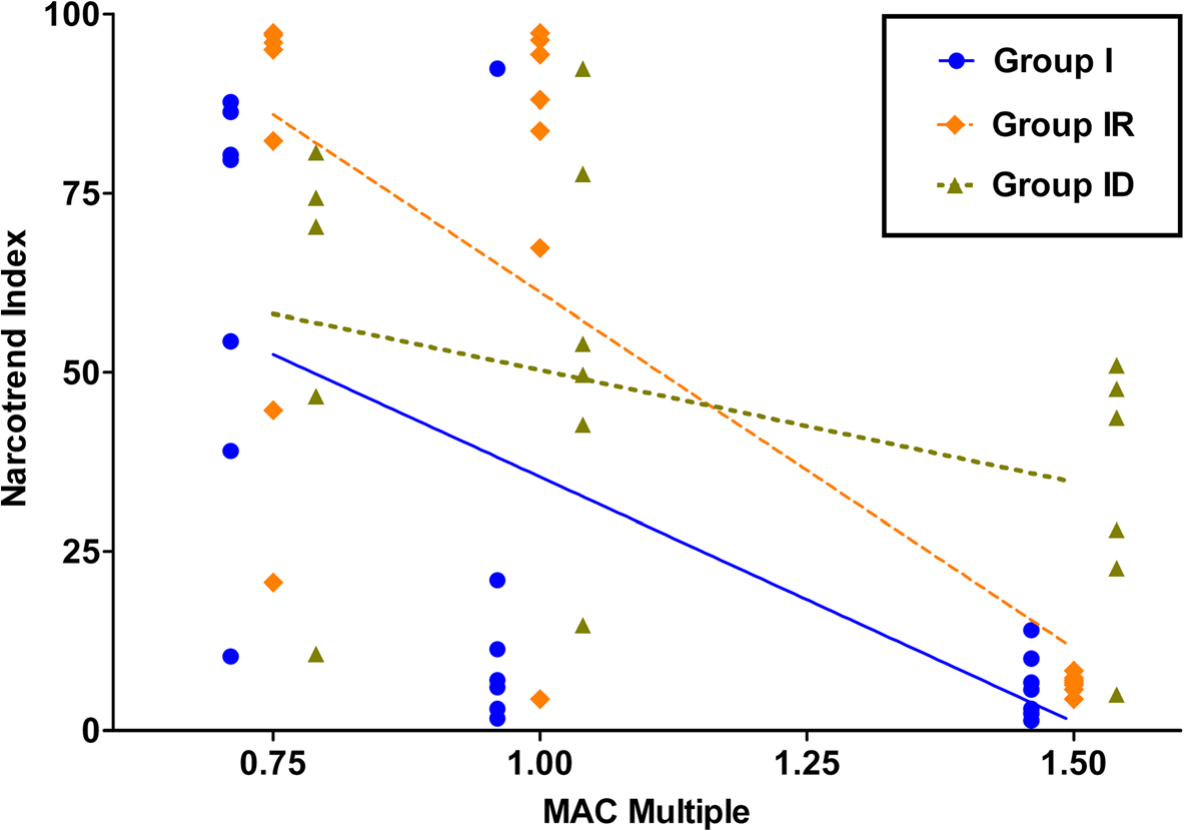
Narcotrend Index (NI) correlation with MAC. NI values of 7 cats (6 in group ID) determined with isoflurane alone (group I, triangles), isoflurane and a constant rate infusion of remifentanil (18 μg/kg/h IV; group IR, diamonds) and with isoflurane and a constant rate infusion of dexmedetomidine (3 μg/kg/h IV; group ID, circles) at different MAC levels (0.75, 1.0 and 1.5 MAC). Prestimulation values were used for correlation analysis. The correlation coefficients for NI were − 0.68 (p = 0.0007) in group I, − 0.67 in group IR (*p* = 0.001) and − 0.34 in group ID (*p* = 0.1659) with increasing MAC level. The slopes of the best-fit linear regression lines were − 68.49 (r^2^ = 0.41, p = 0,0019) in group I, −99.39 (r^2^ = 0.58, p = < 0.0001) in group IR and − 31.35 (r^2^ = 0.16, *p* = 0.97; not significant) in group ID

The Narcotrend Index (NI) showed a strong inverse correlation with MAC multiples in group I (r = − 0.68, *p* = 0.0007) and group IR (r = − 0.66, *p* = 0.001) as shown in Fig. 1. Moderate to strong correlation with MAC was also found in α band presence in group I (r = 0.56 *p* = 0.0085) and group IR (r = 0.63, *p* = 0.0024), as well as in SEF in group I (r = − 0.62 *p* = 0.0029) and in group IR (r = − 0.75, p = < 0.0001). MF was moderately correlated with MAC in group I (r = − 0.45, *p* = 0.0393), whereas, no correlation was found for MF and MAC in group ID.

**Fig. 2:**
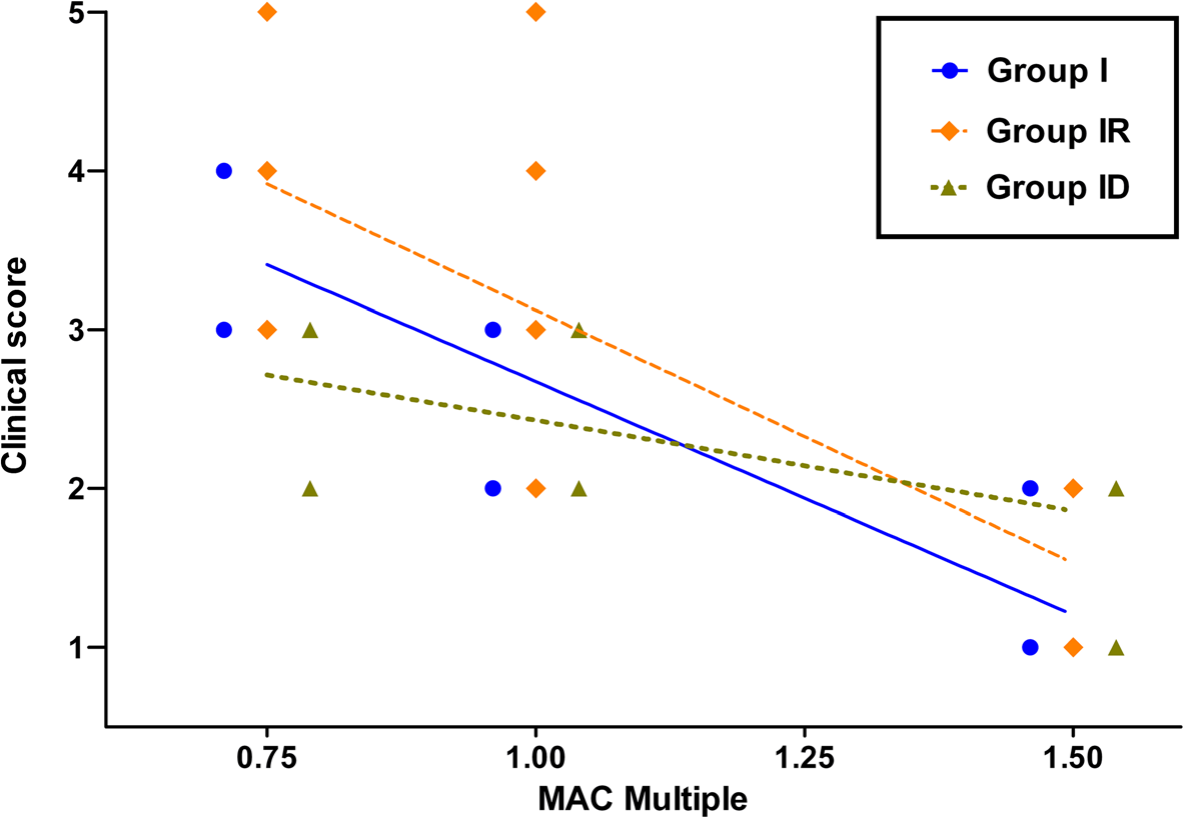
Clinical Score correlation with MAC. Clinical score values of 7 cats (6 in group ID) determined with isoflurane alone (group I, triangles), isoflurane and a constant rate infusion of remifentanil (18 μg/kg/h IV; group IR, diamonds) and with isoflurane and a constant rate infusion of dexmedetomidine (3 μg/kg/h IV; group ID, circles) at different MAC levels (0.75, 1.0 and 1.5 MAC). Prestimulation values were used for correlation analysis. The correlation coefficients for the clinical score were − 0.89 (p = < 0.0001) in group I, − 0.73 in group IR (*p* = 0.0002) and − 0.59 in group ID (*p* = 0.01) with increasing MAC level. The slopes of the best-fit linear regression lines were − 2.94 (r^2^ = 0.76, p = < 0.0001) in group I, − 3.18 (r^2^ = 0.5658, p = < 0.0001) in group IR and − 1.14 (r^2^ = 0.38, *p* = 0.0064) in group ID

The clinical score showed an overall stronger correlation with MAC multiples than any EEG parameter in all groups (Fig. 2). The strongest correlation was found in group I (r = − 0.89, p = < 0.0001, r^2^ = 0.76), followed by group IR (r = − 0.73, *p* = 0.0002, r^2^ = 0.56) and ID (r = − 0.59, *p* = 0.01, r^2^ = 0.38). Noxious stimulation led to a significant increase of the score at 0.75 MAC (*p* = 0.0305) and 1.0 MAC (*p* = 0.0177) when isoflurane was administered alone. The score distinguished significantly between 0.75 MAC and 1.5 MAC in group I (p = < 0.0001), whereas differentiation of 0.75 MAC and 1.5 MAC as well as 1.0 MAC and 1.5 MAC was possible (*p* = 0.0036) in group IR, but not in group ID.

## Discussion

In the present study, a correlation between EEG parameters derived by the Narcotrend system and anaesthetic depth defined by isoflurane MAC multiples was confirmed in cats. The cerebrocortical activity of cats differed among the treatment groups as well as among MAC multiples. The greatest overall EEG depression with increasing MAC was found in group I, which corresponds to the results of a preceeding beagle study [18]. Isoflurane depresses EEG parameters dose-dependently and causes increasing amounts of burst suppressions at higher isoflurane concentrations, but also at clinical useful doses [19]. Studies in humans and dogs anaesthetized with isoflurane report an inverse correlation of increasing isoflurane concentrations with decreasing overall amplitude and the onset of burst suppression patterns after an initial EEG activation (desynchronization) [20, 21]. The correlation of the NI with MAC multiples in the present study suggests a strong hypnotic effect of isoflurane in cats.

Noxious stimulation did not result in NI changes throughout all groups in our study except in group I at 1.0 MAC. At higher isoflurane concentrations, the lack of response might be caused by an isoflurane-induced block of the nociceptive transmission from the spinal cord to the brain, which has been previously reported [22, 23]. At lower isoflurane concentrations, this effect is unlikely to occur; still, no significant NI changes were observed at 0.75 MAC in the cats, which could be explained by the high pre-stimulation NI at this anaesthetic state. The NI response at 1.0 isoflurane MAC is indicative of a medium anaesthetic state without blocked nociceptive transmission, but deeper hypnosis than at 0.75 MAC. In our study, we also observed other EEG parameter changes than the NI after noxious stimulation, but neither the changes themselves nor the comparison of the difference values followed a consistent pattern making it valuable for improved anaesthetic monitoring.

Other studies examined feline BIS at different isoflurane MAC multiples, resulting in a linear inverse correlation of BIS with MAC [11, 12]. However, the BIS in one of these studies [11] was relatively high, whereas in the other study [12], it was lower than expected even at low isoflurane concentrations. Similar to our study, both studies used subdermal needles to obtain the BIS. The NI, ranging from 100 to 0, is subdivided in several stages ranging from A (awake) to F (isoelectricity). In humans, stage D (NI 37–64) reflects an anaesthetic plane which is considered to be medium (“general anaesthesia”) [8]. The NI in our study was higher at 0.75 MAC in group I and IR, maybe indicating a better accordance of the Narcotrend’s algorithms with feline EEG patterns at lighter anaesthetic states compared to the BIS studies. But there are also different experimental settings which have to be considered. For example, in one of the studies [11] visceral stimulation was used, whereas in the other study the cats were stimulated by the tail clamping method [12].

The addition of dexmedetomidine, an α_2_adrenoceptor agonist which creates its hypnotic effects by occupying receptors in the locus coeruleus in the brain [24] and analgesic and muscle relaxing effects via the dorsal horn of the spinal cord, led to a strong isoflurane MAC sparing effect as shown in previous studies [25–27]. The NI did not correlate well with different MAC multiples in the presence of dexmedetomidine and the NI values at 1.0 and 1.5 MAC were high, compared to the other groups. A MAC sparing effect of a drug can be related to hypnosis, analgesia or muscle relaxation. Due to the distinct MAC sparing effect of dexmedetomidine low isoflurane concentrations were used, and it is likely that the analgesic and muscle relaxing features of dexmedetomidine concealed the hypnotic component of dexmedetomidine, even if a synergistic effect with isoflurane [28] might have been present. Still, MAC and NI are not necessarily connected, because MAC is a measurement of immobility. It is influenced by analgesia via spinal pathways, whereas the EEG (and therefore in this study the NI) reflects the activity of the brain and the hypnotic state only.

Contrary to beagle dogs [18], noxious stimulation did not lead to marked arousal reactions in group ID. In cats, similar EEG findings during halothane and dexmedetomidine anaesthesia have been described, but with different levels of consciousness and with a more pronounced clinical response to noxious stimulation during dexmedetomidine anaesthesia [29]. In some cases at lower isoflurane concentrations in our study, very short periods of arousal (with a length of less than 10 s) with immediate re-adjustment to the prestimulation status were obvious clinically as well as in short NI increases, but these were not detected statistically because of the analysis of one-minute-epochs.

Remifentanil is a potent opioid and its analgesic and immobilizing effects in cats have been previously described [30–32]. The dosage used in our study was based on clinical experience and published work. In cats, opioid administration often leads to central stimulation, euphoria and arousal [33, 34], which is most likely the cause of the lack of a significant MAC sparing effect of remifentanil in our study and also explains the slightly higher isoflurane requirements of two cats in group IR after remifentanil administration. The correlation of NI with MAC in group IR was strong and close to the results in group I. This contrasts results obtained in beagle dogs, which did show a stronger MAC sparing effect and poorer correlation of NI with MAC multiples when remifentanil was added to the protocol [18]. Since the NI mainly displays the hypnotic component of anaesthesia [35], this indicates a similar hypnotic state in group I and IR, explainable by very similar isoflurane concentrations.

One MAC is defined as the end-tidal concentration of an anaesthetic agent that just prevents gross muscular movement in response to a maximally nociceptive painful stimulus in 50% of the patients [36]. However, even if this standard method [37] helps to obtain an objective and comparable state of immobility, it is still questionable if a similar levels of anaesthetic depth is achieved. A problem is that there is still no clear definition of anaesthetic depth. Hypnosis, immobility and adequate analgesia are needed for most surgical procedures. Since remifentanil and dexmedetomidine both have proven analgesic as well as hypnotic effects, and both alter EEG parameters [29, 38, 39], the influence of each aforementioned property, however, can only be estimated.

Burst suppression patterns and periods of EMG activity were observed in some cats in our study. These were considered not to influence the NI [8, 40] because of its underlying algorithm, which includes burst suppression and is not displayed during EMG activity. Still, this algorithm was developed based on human EEG studies and possible mismatches with feline EEG parameters need to be kept in mind.

Increases of heart rate or blood pressure after noxious stimulation are signs of activation of the autonomous nervous system. Those autonomous responses were observed mainly during sessions with lower MAC multiples, but also in some cats at 1.5 MAC in group ID, in the absence of EEG responses. Hemodynamic responses are from subcortical origin and can occur independently from a conscious pain experience [41]. It is to mention that we did not obtain the MAC BAR (block adrenergic response) in this study, which is the minimal alveolar concentration of an inhalant anaestetic agent that is able to suppress ANS responses to pain stimulation [42]. Compared to the standard MAC (or MAC_50_), more anaesthetic agent is needed for ANS suppression.

Generally, the clinical score used in this study correlated better with MAC multiples than any of the EEG parameters. Still, as mentioned above, EEG parameters and the NI reflect mainly the hypnotic state of the patient. Since the inhibition of movement and reflexes is processed in the subcortical part of the nervous system [43], it is likely that our clinical score was able to detect smaller differences at lighter anaesthetic planes without changes in the state of consciousness. Furthermore, only 20–45% of the volatile anaesthetic is needed to reach unconsciousness and amnesia, compared to the amount of anaesthetic which provides suppression of purposeful movement [44]. The score we used in this study is displayed as a single number that combines several clinical parameters. There was some individual variation in the appearance of the single clinical parameters among the cats with increasing or decreasing MAC. The mean of these different values may not necessarily reflect the anaesthetic state correctly, but taking more than one parameter into account helps to prevent misinterpretation of the clinical situation.

As mentioned before, similar to the study performed with beagle dogs [18], the pre-stimulation EEG values were collected for 1 min before noxious stimulation, and the same epoch length was used for analysis of the post-stimulation phase, starting directly after end of stimulation. Due to the fact that some cats tended to show delayed reaction to noxious stimulation, epoch lengths up to 3 min have been examined in this study, but since most of these longer epochs only revealed re-adjustment to the prestimulation values, or, in some cases, did not show difference to the shorter epochs, only the 1 min epochs were used for further statistics. It is also to mention that longer epochs would not be suitable in a clinical setting. During surgical procedures, it is often necessary to detect changes in the anaesthetic state more quickly.

### Limitations

The low number of cats and the wide range of obtained individual NI values among the cats at the same MAC is a possible limitation of our study and might have lead to underpowered results.

The MAC multiples 0.75, 1.0 and 1.5 were chosen for practical reasons. Using 0.5 MAC was not possible, because the cats woke up as shown in pre-trials. Also, larger MAC intervals might have revealed more prominent differences in EEG variables.

Another considerable limiting factor is the variation of the MAC levels between the groups. Only the amount of isoflurane was changed from 1.0 MAC to 0.75 MAC or 1.5 MAC, but the CRI in groups ID and IR remained the same. This might have led to different levels of hypnosis despite the same MAC when comparing the groups.

The neurologic examinations of the cats did not show signs of disease. Still, no further examinations like brain MRI was performed, and therefore neurologic disorders with influence on our measurements cannot be completely excluded.

## Conclusions

The EEG parameters derived from the Narcotrend Monitor show correlation to isoflurane MAC multiples in cats, but the specific anaesthetic protocol and especially the addition of dexmedetomodine have great influence on the reliability. The Narcotrend Monitor can be used as an additional tool to assess anesthetic depth in cats.

## Methods

### Animals

Seven experimental cats were used. All cats were adult European Domestic Short Hair breed, two female, one female-spayed, five male-neutered. They were provided by the Institute for Parasitology of the University of Veterinary Medicine Hannover, Foundation, Bünteweg 9, 30,559 Hannover, Germany.

Mean age ± SD was 5.6 ± 3.0 years and mean body weight was 4.5 ± 0.96 kg. All animals underwent a physical and neurologic examination and hematology and blood biochemistry were performed. The cats were held off food for 8 h, but water was offered until 1 h prior to beginning of the experiment. Only one animal was tested per day, so anaesthesia could be started at the same time of the day in all cats, in order to exclude influences of a circadian rhythm. After recovery from the anaesthesia, the cats were transferred back to their familiar housing. No cats were euthanized.

The study was performed in accordance with the German animal protection law after review and approval by the ethical committee for animal experimentation of the Federal State Office for Consume Protection and Food Safety of Lower Saxony, Germany (approval number: 33.12–42,502–04-10/0102).

### Experimental design

This study was done in a prospective and complete cross-over design. Each cat was assigned to 3 experimental treatment groups defined by different anaesthesia protocols. A wash-out period of at least 8 days was set between the experiments, and the individual treatment order for each cat was randomized. The MAC was determined individually by supramaximal electrical stimulation, and 1.0 MAC was the first anaesthetic plane on which measurements were performed. Afterwards, further MAC multiples (0.75 MAC and 1.5 MAC) were investigated in randomized order. The same stimulation protocol as used for the MAC determination was carried out at each of the 3 MAC multiples.

### Anaesthesia

In group I, anaesthesia was performed only with isoflurane, whereas in group IR a CRI of remifentanil (18 μg/kg/h IV) was added, and group ID the cats received isoflurane and a CRI of dexmedetomidine (3 μg/kg/h IV). Isoflurane was administered in 100% oxygen. Saline solution (0.9% NaCl) was used to dilute remifentanil and dexmedetomidine so that the CRI could be set at a rate of 5 ml/kg/h. The cats in group I received a CRI of pure saline solution at the same rate.

### Instrumentation

Prior to each experiment, an intravenous catheter was placed in a saphenous or cephalic vein. Anaesthesia was induced by isoflurane inhalation in an induction chamber (5 vol% isoflurane in 100% oxygen at a flow rate of 5 L/min) until righting reflex loss. Mask induction followed until endotracheal intubation could be performed. The cats were positioned in right lateral recumbency and were connected to an anaesthetic circle system. The individual CRI was started, and the end-tidal isoflurane level was set slightly above the estimated 1.0 MAC. Isoflurane and CO_2_ measurements were made via infrared spectroscopy.^1^ A reference gas (5.00% CO_2_, 33.0% N_2_O, 2% desflurane and N_2_ as balance gas) was used for calibration of the multiparameter monitor prior to each experiment. The same monitor was used to measure SpO_2_. Artificial ventilation provided eucapnia (35–45 mmHg end-tidal CO_2_). An esophageal probe was used for body temperature measurements, and temperature was held in physiological ranges by use of a warm air blanket. Systolic arterial blood pressure was measured via Doppler technique at a metatarsal artery or the coccygeal artery.

The nociceptive stimuli were given by a square pulse stimulator^2^ (settings: 50 V, 50 Hz and 10 ms) which was connected to 2 isolated stimulation electrodes. These were placed subcutaneously in the middle aspect of the right medial ulnar region, with a distance of 4–5 cm between the electrodes.

The raw EEG signal was recorded and processed with a Narcotrend Monitor^3^ by standard needle electrodes using a single-channel registration. The recording electrodes were subcutaneously placed as follows: one electrode on each side in the temporal region, in the middle of an imaginary line between the lateral canthus and the ear, and the reference electrode, also serving as the ground of the amplifier, was placed on the bridge of the nose. Correct needle placement was checked by automatic impedance measurements. Impedance had to be below 6 kΏ otherwise needle placement was changed. The EEG was continuously recorded and processed, and data were stored by the monitor for off line analysis.

Additionally, for another part of the study [45], four ECG surface electrodes were placed palmar and plantar on the paws, or, if the signal was too low, laterally on both sides of the chest.

After each experiment, anaesthesia was discontinued and all parts of the instrumentation were removed from the cat. To prevent possible inflammation or pain in the stimulation area, all cats received a single dose of meloxicam (0.1 mg/kg) subcutaneously after each experiment.

### MAC determination

The instrumentation period was set to 60 min, and the individual MAC was determined afterwards. A standardized stimulation protocol [46], was applied. This included two single stimuli and two continuous stimuli of 3 s, with pauses of 5 s. If a positive reaction was observed, the stimulation protocol was stopped immediately. Gross head or leg movement (but not movement of the stimulated leg) or tail movement was considered a positive reaction. Swallowing, eye movement, tongue or ear movement, spontaneous breathing efforts or chewing was defined as a negative. In the initial phase of this study, some cats reacted to the stimuli with delay. Therefore, a period of 1 min after stimulation was allowed to display a positive reaction. Later reactions were defined as negative. Using the bracketing method [47], the ET_ISO_ level was lowered or raised 0.2 vol% depending on the cat’s reaction, followed by another equilibration phase of 20 min after reaching the desired ET_ISO_ level. In the final step, the ET_ISO_ level was changed 0.1 vol%, and the individual 1.0 MAC was stated as the arithmetic mean of those ET_ISO_ values that just prevented or permitted a positive reaction, respectively.

### EEG measurements

At each experiment, EEG was measured continuously at 128 samples per second with a 12-bit resolution. The amplifier’s filter settings were 0.5 to 45 Hz, and they were combined with a supplemental 50-Hz notch filter. The signal was automatically processed via Fast Fourier transformation of 2-s segments, and the parameter values were provided as means of 10 consecutive 2-s segments (displayed as 20-s epochs). Six different frequency bands were defined as follows: δ = 0.5 to 3.5 Hz, θ = 3.5 to 7.5 Hz, α = 7.5 to 12.5 Hz, and β = > 12.5 Hz. The EEG was visually checked for temporary signal absence or artifacts before further analysis. EMG activity and burst suppression patterns were noted, but not excluded. The frequency band values, 95% spectral edge frequency (SEF), median frequency (MF) and the Narcotrend index (NI) were provided by the Narcotrend for further offline analysis^4^. Epochs with mean values out of 1, 2 and 3 min before and after stimulation were used for analysis.

### Clinical scoring of anaesthetic depth

Adapted from a modified Guedel-scheme, a clinical score ranging from “very deep” (1) to “very light” (5) was evaluated before and after stimulation during the same time epochs as used for the EEG measurements. Pupil position and size, eyeball movement, eye reflexes, heart rate and blood pressure, nictitating membrane position, spontaneous breathing efforts, jaw tone, spontaneous movement and swallowing were included in the scoring system (Table 1).

**Table 1:** Clinical score of anaesthetic depth in cats

Anaesthetic plane	Clinical Score	Pupil position/size	Eye reflex (P=Palpebral, C=Corneal)	Heart rate, blood pressure, breathing	Third eyelid position	Jaw tone, body/limb movement or swallowing
Very light	5	Central, large or small, moving	P: active C: active	Increased, spontaneous spontaneous breathing attempts	Retracted	Strong
Medium-light	4	Central or ventromedial, large or small,	P: active or mildly depressed C: active	Increased, spontaneous breathing attempts	Retracted or partially prolapsed	Medium
Medium	3	Ventromedial, small or medium	P: depressed C: mildly depressed	Normal	Completely Prolapsed	None or barely
Medium-deep	2	Central, medium	P: depressed or markedly depressed C: depressed	Normal or depressed	Completely or partially prolapsed	None
Very deep	1	Central, large	P: absent C: markedly depressed or absent	Depressed	Partially prolapsed	None

Mean arterial blood pressure > 90 mmHg was defined as “increased”, < 60 as “depressed”. Heart rates between 120 and 180 beats per minute were defined as “normal”, lower values as “depressed” and higher values as “increased”

### Statistical analysis

Commercial software^5^ was used for statistical analysis. Because only 7 cats (6 cats in group ID) were used, the EEG parameter and clinical score values cannot be reliably assessed for normality. Therefore, the non-parametric Wilcoxon signed-rank test for repeated measures was used for comparison of pre- and post-stimulation data. The Friedman test and Dunn’s multiple comparison test were performed for comparison of the pre-stimulation values at different MAC multiples within the same group. Because of missing data of one animal in the dexmedetomidine group, the Skillings-Mack test was used instead of the Friedman test for comparison of pre-stimulation values and pre−/post-stimulation differences between the anaesthetic treatment groups at the same MAC level, followed by Wilcoxon signed rank tests and Bonferroni type I error correction in cases with statistical significance. The correlation of EEG parameters and clinical score values with MAC multiples was assessed by Spearman’s rank correlation and linear regression analysis. The significance level was set as *p* < 0.05.

## Supplementary information

**Additional file 1: Table S1.** EEG parameter values before and after nociceptive stimulation.

## Data Availability

The datasets used and analysed during the current study are available from the corresponding author on reasonable request.

## Abbreviations

CRI: Constant rate infusion
EEG: Electroencephalography
EMG: Electromyography
ET_ISO_: End-tidal isoflurane concentration
I: Isoflurane alone
ID: Isoflurane and dexmedetomidine
IR: Isoflurane and remifentanil
MAC: Minimum alveolar concentration
MF: Median frequency
NI: Narcotrend Index
SEF: Spectral edge frequency

## Footnotes

1 Datex Ohmeda Compact Monitor, GE Healthcare, Fairfield, Connecticut, USA

2 Grass S48 Square Pulse Stimulator, Astro-Med, West Warwick, Rhode Island, USA

3 Narcotrend-Compact, version 5.0, MT Monitor Technik GmbH & Co KG, Bad Bramstedt, Germany

4 NarcoWin, version 1.0, MT Monitor Technik GmbH & Co KG, Bad Bramstedt, Germany

5 GraphPad Prism, version 5.01, GraphPad Software, La Jolla, California, USA
